# High shape and weight importance in adolescence relates to worse body image in the first perinatal period

**DOI:** 10.1016/j.eatbeh.2024.101931

**Published:** 2024-11-19

**Authors:** Lyza Norton, Rebecca L. Emery Tavernier, Katie Loth, Susan Mason

**Affiliations:** a University of Melbourne, School of Population and Global Health, 207 Bouverie Street, Carlton, Victoria 3010, Australia; b Weitzman Institute, 631 Main St., Middletown, CT 06457, United States of America; c University of Minnesota Medical School, Duluth Campus, 1035 University Dr., Duluth, MN 55812, United States of America; d Department of Family Medicine and Community Health, University of Minnesota, 717 Delaware Street SE, Minneapolis, MN 55454, United States of America; e Division of Epidemiology and Community Health, University of Minnesota School of Public Health, 1300 South 2^nd^ Street, Suite 300, Minneapolis, MN 55454, United States of America

**Keywords:** Body image, Adolescents, Pregnancy, Postpartum, Disordered eating

## Abstract

Understanding whether shape and weight importance in adolescence relates to body image during the perinatal period is essential to inform eating disorder prevention and intervention research. We longitudinally examined the relationship between weight and shape importance during adolescence and body image during (1) pregnancy and (2) the first postpartum year. Participants included 554 women from the Life-course Experiences and Pregnancy (LEAP) study, a longitudinal cohort of women participating since adolescence in Project EAT (Eating and Activity in Teens and Young Adults). Participants reported shape and weight importance during adolescence (11–18 years old) for Project EAT and retrospectively recalled body image during and after their first pregnancy for the LEAP study 20 years later. Separate linear regression models estimated the longitudinal associations between adolescent shape and weight importance and pregnancy and postpartum body image. After covariate adjustment, results showed small, positive associations between adolescent shape and weight importance and pregnancy (*B* = 0.07 *95 % CI: 0*.02, 0.11) and postpartum (*B* = 0.10, *95 % CI: 0*.06, 0.14) body image. These findings indicate that high shape and weight importance in adolescence relates to worse body image across the perinatal period. Results highlight the importance of (1) targeting the years prior to adolescence for eating disorder prevention interventions and (2) providing support and interventions for people in the perinatal period, specifically targeting body image.

## 1. Introduction

Understanding risk factors for eating disorders is important for the development of successful health promotion campaigns and prevention interventions (Jacobi et al., 2004). One key risk factor for eating disorders is body dissatisfaction (Barakat et al., 2023). Indeed, the current Diagnostic and Statistical Manual of Mental Disorders (DMS-5-TR) states “undue influence of body weight or shape on self-evaluation” as one of the core diagnostic criteria for both anorexia and bulimia nervosa (APA, 2022). The perinatal period is a particularly vulnerable time for shifts in body satisfaction and represents a critical window for understanding risk factors related to body image concerns.

It has been argued that pregnancy and birth may represent a period where birthing people develop a greater sense of awe for and appreciation of the functionality of their bodies (Fox & Neiterman, 2015). It is plausible that these feelings may then manifest in an improved body image. In keeping with this theory, Loth et al. (2011) examined body satisfaction prior to and during pregnancy and found a significant increase in body satisfaction over this period. Previous qualitative research also supports this argument, highlighting that pregnant people who maintain a focus on their body’s functionality during pregnancy are more protected against body dissatisfaction (Clark et al., 2009). In contrast, in the postpartum period, body dissatisfaction is frequently reported as a major concern for birthing people (Hartley et al., 2018; Hodgkinson et al., 2014), who often report desiring their weight to be lower and feeling distressed over not achieving their desired weight (Hodgkinson et al., 2014). A reoccurring theme in the literature is that birthing people require more support with accurate health information in the postpartum period regarding reasonable expectations for weight loss and body changes after childbirth (Corr et al., 2015; Lovering et al., 2018; Vanderkruik et al., 2022). There is also evidence that birthing people may be especially vulnerable to pressures of the “thin-ideal” in the second half of the postpartum year as time from the birth increases (Lovering et al., 2018), which likely contributes to increased risk for body dissatisfaction.

The shifts in self-evaluation that accompany the dramatic changes in physical appearance during pregnancy and postpartum may be further impacted by earlier beliefs about one’s shape and weight. Shape and weight concerns often develop in adolescence (Calzo et al., 2012; Hoffmann & Warschburger, 2017), with approximately 20 % of adolescents reporting distress regarding weight and shape (Bartholdy et al., 2017), and increase risk for disordered eating over time (Loth et al., 2014). However, understanding of the trajectory of shape and weight importance from adolescence to pregnancy and postpartum in adulthood remains an understudied area for eating disorder prevention and intervention efforts.

Given that shape and weight preoccupation is one of the strongest longitudinal predictors of disordered eating (Askew et al., 2020), understanding how body image tracks over the life course is important for identifying points of intervention to prevent disordered eating. Thus, the current study aims to quantitatively explore body image in birthing people through three critical stages in their life course: adolescence, pregnancy, and postpartum. The primary research questions are: (1) What is the relationship between shape and weight importance during adolescence and body image during first pregnancy? and (2) what is the relationship between shape and weight importance during adolescence and body image during the first 12 months after the first live birth? This research is significant as it represents the first longitudinal exploration of whether adolescent shape and weight importance serves as a modifiable risk factor for perinatal body image.

## 2. Methods

### 2.1. Participants and procedures

The Life-course Experiences and Pregnancy (LEAP) study collected retrospective survey and medical record data on pregnancy characteristics among women participating since adolescence in Project EAT (Eating and Activity in Teens and Young Adults), a Minnesota-based prospective cohort study of eating, activity, and weight-related health (Neumark-Sztainer et al., 2018). The original Project EAT survey included 4746 adolescents, of whom 50 % (*n* = 2373) were girls. Details of LEAP are described elsewhere (Mason et al., 2024). Briefly, in 2019, participants from Project EAT were invited to fill out the LEAP survey if they had responded to either of the two prior waves of Project EAT surveys, had a US-based mailing address, and identified as women (*n* = 1251). The LEAP survey collected retrospective self-report data on participants’ reproductive histories, including pre-pregnancy weight and body image during and after pregnancy, for each of their live births. LEAP data were linked with data on weight-related risk factors, including shape and weight importance, collected during the first Project EAT survey when participants were aged 11–18 years (EAT-I; 1998–1999). Of the 1251 women invited to participate in LEAP, 977 (78 %) completed the survey between July 2019 and July 2020. Of these, 656 (67 %) reported at least one live birth. At the time of survey completion, LEAP participants were an average of 36.4 years of age (*SD* = 1.5) and had completed the EAT-I survey when they were 15.1 years of age (*SD* = 1.5). The current analysis was restricted to the first live birth for each woman, meaning that subsequent live births and any pregnancies resulting in miscarriage or stillbirth were not included. The University of Minnesota Institutional Review Board Human Subjects Committee approved all protocols implemented in the LEAP Study.

### 2.2. Measures

#### Pregnancy and postpartum body image:

On the LEAP survey, participants retrospectively recalled their body image during their pregnancy with their first live birth and in the 12 months after their first live birth using five items adapted from the Body Image in Pregnancy Scale (BIPS) (Watson et al., 2017). Using a 5-point Likert-scale ranging from “Never” to “Always,” participants reported the extent to which they (1) worried about their weight, shape, or size, (2) felt unattractive because of their weight, shape, and size, (3) were more concerned about how their body functioned than how it looked (reverse coded), (4) felt happy with how their body functioned (reverse coded), and (5) felt happy with their weight, shape, and size (reverse coded) during their pregnancy and postpartum. An overall body image score was calculated for both the pregnancy and postpartum periods by averaging all five items, with higher scores indicating worse body image. Cronbach’s alphas were 0.71 for pregnancy and 0.70 for postpartum, indicating adequate internal reliability.

#### Adolescent shape and weight importance:

At EAT-I, Participants reported the importance of shape and weight using an adapted item from the Questionnaire on Eating and Weight Patterns-Revised (QEWP-R, Yanovski, 1993). Participants were asked, “During the past six months, how important has your weight or shape been in how you feel about yourself?” Participants selected from four response options indicating that weight and shape (1) were not very important in, (2) played a role in, (3) were among the main things, and (4) were the most important things affecting how they felt about themselves. Following the Diagnostic and Statistical Manual of Mental Disorders-5 cut-off for eating disorders (Yanovski et al., 2015), participants were dichotomized into two groups: (1) those reporting that shape and weight were not very important or played a minimal role in how they felt about themselves and (2) those reporting that shape and weight were among the main things or the most important things affecting how they felt about themselves.

#### Covariates:

Race and socioeconomic status (SES) were self-reported at the EAT-I assessment and used as covariates. SES was based on parents’ education, defined as the highest level of education of either parent, with missing or implausible values imputed or corrected using information on eligibility for public assistance, free or reduced-cost school meals, and parental employment status (Sherwood et al., 2009). We elected to control for SES at EAT-I rather than that reported during the LEAP survey because SES in childhood is known to directly impact health outcomes across the lifespan, including BMI (Merrick, 2019; Schroeder et al., 2021), which itself alters risk for body image concerns (Mintem et al., 2015). Further, SES in adulthood may be influenced by adolescent factors including body image, and thus may mediate the association between adolescent shape and weight concerns and perinatal body image rather than simply acting as a confounder of this relationship. Consequently, controlling for adult SES measured during the LEAP survey has the potential to adjust away effects that we are trying to measure and may introduce unnecessary bias (Wang et al., 2017).

In addition, participant age and pre-pregnancy BMI for their first live birth were derived from the LEAP survey and used as covariates. With regard to pre-pregnancy BMI, the LEAP survey asked participants to estimate their weight just before their first live birth. Pre-pregnancy BMI for the first live birth was calculated from this reported pre-pregnancy weight and women’s self-reported height on EAT-IV (age 25–36). If height was missing on EAT-IV (*n* = 52), height reported on either LEAP (*n* = 48) or EAT-III (n = 4) was used instead. In a validation subsample of LEAP participants (*n* = 159), pre-pregnancy BMI was correctly self-reported among a majority of participants (72 %) when compared to pre-pregnancy BMI extracted from medical records, indicating adequate validity of self-reported pre-pregnancy BMI in this cohort (Farkas et al., 2024).

### 2.3. Statistical analysis

Analyses were conducted using IBM SPSS Statistics for Windows, Version 26.0 (IBM, Armonk, NY). Participants with missing shape and weight importance (*n* = 17), pregnancy body image (*n* = 6), postpartum body image (*n* = 40), and covariate (*n* = 39) data were excluded, resulting in an analytic sample size of 554 participants. To examine the association between adolescent shape and weight importance and later perinatal body image, a series of linear regression models were built. Separate models were run for each outcome (i.e., pregnancy and postpartum body image) regressed on adolescent shape and weight importance, and unadjusted and adjusted models were estimated. Adjusted models included maternal age at first live birth, pre-pregnancy BMI for first live birth, structurally racialized groups^1 and 2^ (6 dummy coded variables, with White coded as the reference group), and SES (4 dummy coded variables, with high SES coded as the reference group). Since pre-pregnancy BMI could plausibly be a mediator on the pathway from adolescent shape and weight importance to body image during the perinatal period, adjusted models were initially run with and without adjustment for pre-pregnancy BMI. However, because adjustment for pre-pregnancy BMI did not meaningfully change the overall pattern of results, only the fully adjustment models are presented herein. Continuous predictors (i.e., age and pre-pregnancy BMI) were grand mean centered to facilitate interpretation. Given growing consensus about the limited information hypothesis testing provides as well as the harms of its widespread use (Amrhein et al., 2019; Wasserstein & Lazar, 2016), we do not report *p*-values. Instead, we provide 95 % CIs as a measure of the precision of our estimates.

## 3. Results

### 3.1. Sample characteristics

As shown in Table 1, a majority of participants identified as White and reported having a middle to high-middle socioeconomic status. Most participants placed high importance on their body shape and weight at the EAT-I assessment. At the time of the first live birth, participants were an average of 27.2 (*SD* = 5.1) years old, with an average pre-pregnancy BMI of 25.4 (*SD* = 5.9). Average scores on the pregnancy and postpartum body image measures were in the low to moderate range, indicating some body image concerns during these periods though not high concerns.

### 3.2. Association between adolescent shape and weight importance and perinatal body image

In the unadjusted model, there was a small positive association between adolescent shape and weight importance and pregnancy body image (*B* = *0*.08, *95 % CI:* 0.04, 0.13), indicating that participants with high shape and weight importance in adolescence reported worse body image in pregnancy when compared to those with low shape and weight importance in adolescence. As shown in Table 2, this effect remained after adjusting for sociodemographic factors and pre-pregnancy BMI, though it was slightly attenuated. More specifically, after adjusting for covariate effects, every one-unit increase in adolescent shape and weight importance related to a 0.07 unit (*95 % CI:* 0.02, 0.11) increase in poorer pregnancy body image, which is equivalent to 0.13 standard deviations.

A similar pattern emerged for postpartum body image. In the unadjusted model, shape and weight importance positively related to postpartum body image (*B* = *0*.12, *95 % CI:* 0.07, 0.16), such that participants with high shape and weight importance in adolescence reported worse body image in the 12 months following their first live birth when compared to those with low adolescent shape and weight importance. This effect was retained after adjusting for covariates (see Table 2). Specifically, for every one-unit increase in adolescent shape and weight importance, there was a 0.10 unit (*95 % CI:* 0.06, 0.14) increase in poorer postpartum body image, the equivalent of 0.18 standard deviations.

With regard to covariate effects, participants who identified as Black/African American reported more positive body image in pregnancy compared to their White peers, after adjusting for their adolescent body image, though this effect was not replicated postpartum. Similarly, increases in age were associated with a more positive body image in pregnancy but not postpartum. In addition, individuals of low-middle to high-middle socioeconomic status generally reported worse body image in pregnancy and postpartum compared to those of high socioeconomic status. Finally, pre-pregnancy BMI was the strongest predictor of both pregnancy and postpartum body image, with higher BMI relating to worse body image across the perinatal period.

## 4. Discussion

This unique study aimed to examine how adolescent perception of shape and weight importance relates to body image in women through two crucial stages of the life course: (1) during their first pregnancy and (2) the first 12 months after their first live birth. Our results highlight that placing a high importance on shape and weight in adolescence is associated with poorer body image in both pregnancy and postpartum when adjusting for relevant covariates. The overall effect sizes for both were small, indicating shape and weight concerns in adolescence may be just one factor influencing the complex and significant trajectory of body image for birthing people during the perinatal period. Indeed, previous research has identified other notable factors that influence body image during the perinatal period, including depression, trait anxiety, BMI and lifetime history of mood disorders (Roomruangwong et al., 2017). Our findings further indicate that demographic factors like racialized identity, age, and socioeconomic status may also influence perinatal body image. Although additional probing into these demographic differences is beyond the scope of this paper, the social factors driving variability in perinatal body image warrant further investigation.

This study joins a growing body of literature focused on understanding perinatal body image. Consistent with the present findings, Rallis et al. (2007) found birthing people to have poorer body image in the postpartum period compared to late pregnancy, with 6 months postpartum being the time of greatest body concern. Additional research has found that different facets of body image change over the course of pregnancy (Linde et al., 2022), with concerns of overall appearance decreasing as pregnancy progresses while dissatisfaction with specific body parts increase. Although a small number of studies have examined physical and psychosocial predictors of perinatal body dissatisfaction (Linde et al., 2020; Roomruangwong et al., 2017), our findings are the first to demonstrate that distal factors like adolescent shape and weight concerns relate to poorer body image across pregnancy and the postpartum. Given that perinatal body image concerns are associated with dietary restraint (Duncombe et al., 2008; Shloim et al., 2015) and binge eating (Linde et al., 2022) in pregnancy as well as increased risk for postpartum depression and anxiety (Chan et al., 2020), our findings underscore the importance for early intervention and prevention efforts.

In particular, our study highlights three potential points across the life span to focus future interventions: (1) prior to adolescence, (2) pregnancy and (3) postpartum. Two-thirds of the adolescents in our sample were found to already place high levels of importance on their shape and weight, which is consistent with previous work showing that many of the attitudes people develop about their bodies are formed in the years preceding adolescence (Damiano et al., 2015). As such, it can be argued that these early years are an essential target for primary prevention initiatives. Over the last decade, universal prevention research targeting the reduction of risk factors for eating disorders in the early years has started to gain interest (Chua et al., 2020). This investment is warranted given that children as young as 5 years old partake in “dieting” (Damiano et al., 2015). A recent systematic literature review explored the effectiveness of universal eating disorder prevention interventions in improving body image in children and adolescents (5–17 years; Chau, 2020). The review found that, out of the 22 eligible trials, only 5 were targeting children under 10 years of age and the majority were school-based initiatives. Promisingly, emerging prevention programs are increasingly targeting younger age ranges using developmentally appropriate theories in an array of different environments (Hart et al., 2016; Jansen et al., 2018; Norton et al., 2023). Considering the high levels of importance adolescents are placing on their shape and weight, and the fact that this type of body preoccupation persists across the life course (Tiggemann & Lynch, 2001), and is one of the most consistent longitudinal predictors of future disordered eating symptoms (Askew et al., 2020), a much larger investment in prevention interventions in early childhood appears justified.

It is also necessary to more adequately support people with body image concerns throughout the vulnerable perinatal period. Recognizing the importance of the entire perinatal period, the concept of a Perinatal Maternal Health Promotion Model has been proposed (Fahey & Shenassa, 2013). Such a model asserts that health goes beyond the absence of medical complications and encompasses four key health-promoting skills, including (1) mobilizing social support, (2) self-efficacy, (3) positive coping strategies and (4) realistic expectations, in both pregnancy and postpartum. Given the findings from our study and the critical repercussions of poor body image for birthing people, an investment in operationalizing such a model is warranted. In keeping with this model, Papini et al. (2022), evaluated the efficacy of a three-week self-compassion and meditation intervention for improving body image during pregnancy and postpartum. Results found those who completed the intervention reported lower body dissatisfaction and body shame, from baseline to post-intervention. However, this study was a pilot study, conducted with small numbers and a dropout rate of 39 %, highlighting that further research is required. Qualitatively exploring birthing people’s views and needs related to body image would be a positive step to co-designing future interventions (Slattery et al., 2020).

There are several limitations to this study. Caution is required when considering the generalizability of the findings as the majority of participants were from middle to high-middle socioeconomic status childhoods and identified as White. Additionally, there was significant attrition across all waves of Project EAT, from which participants were recruited for the LEAP study. Although 78 % of the 1251 eligible women invited to participate in LEAP completed the survey, there remains a possibility that selection bias exists in this sample. Indeed, a respondent’s probability of participating in a study is correlated with their interest in the topic (Lavrakas, 2008); hence, we acknowledge the data do not represent the entire cohort. In addition, participants retrospectively recalled their body image during their first pregnancy and postpartum period, which may have resulted in recall bias. Moreover, the adolescent shape and weight importance and perinatal body image variables examined in this study were assessed using a subset of items from validated tools rather than the whole measures. Although subsets of scales are commonly used when conducting large epidemiologic surveys to gain a general assessment of population health and allow for ease of identification of high-risk individuals, they do not allow for detailed clinical assessments and may result in unintentional measurement errors. As such, future research is needed to extend these findings using more extensive questionnaire measures. Despite these limitations this study does have several strengths, including a large sample of community participants. The key strength of this research is it substantially increases our understanding of body image from adolescence through pregnancy and the postpartum period. It is the only longitudinal study to examine this important and under-researched area.

## 5. Conclusion

This is the first study to longitudinally examine the association between adolescent shape and weight importance and body image in the perinatal period. Findings indicate that high importance of shape and weight in adolescence had a modest association with poorer body image in both pregnancy and postpartum, after adjusting for relevant covariates. Such findings highlight two key areas for future research and clinical implications: (1) the importance of early body image prevention interventions targeting the years before the adolescent period and (2) the importance of providing support for birthing people in the perinatal period; for example, through the provision of programs focused on body expectations in the postpartum period. While environmental, health promotion and policy regulation all play a role in enhancing the way people feel about their bodies, our study underscores the need to also support individual families to enhance their relationships with food and bodies, preferably starting at an early age.

## Figures and Tables

**Table 1:** Sample characteristics (*N* = 554).

	Mean	SD

Pregnancy body image	2.38	0.77
Postpartum body image	2.84	0.80
Age	27.24	5.08
Pre-pregnancy BMI	25.36	5.85

	n	%

Shape and weight importance*		
High	366	66.1 %
Low	188	33.9 %
Racialized groups*		
White	374	67.5 %
Black/African American	34	6.1 %
Hispanic/Latinx	18	3.2 %
Asian American/Pacific Islander	86	15.5 %
Multi or other race	42	7.6 %
Socioeconomic status*		
Low	79	14.3 %
Low-middle	82	14.8 %
Middle	140	25.3 %
High-middle	169	30.5 %
High	84	15.2 %

*Note:* BMI = body mass index.

* Variable measured at EAT-I.

**Table 2:** Multiple linear regression models evaluating the association between adolescent shape and weight importance and body image in pregnancy and postpartum.

	Pregnancy Body Image			Postpartum Body Image		
		
	*β*	*B*	*95 % CI for B*	*β*	*B*	*95 % CI for B*

Shape and weight importance*						
High	0.13	0.07	0.02, 0.11	0.18	0.10	0.06, 0.14
Low	–		–	–		–
Racialized groups*						
White	–		–	–		–
Black/African American	−0.09	−0.29	−0.56, −0.02	−0.06	−0.21	−0.48, 0.07
Hispanic/Latinx	0.04	0.08	−0.10, 0.26	−0.03	−0.08	−0.26, 0.10
Asian American/Pacific Islander	−0.03	−0.02	−0.09, 0.05	0.06	0.05	−0.02, 0.11
Multi or other race	−0.01	−0.01	−0.05, 0.03	0.01	0.01	−0.04, 0.05
Socioeconomic status*						
Low	0.08	0.17	−0.08, 0.42	0.09	0.20	−0.06, 0.46
Low-middle	0.10	0.10	−0.01, 0.22	0.13	0.15	0.03, 0.26
Middle	0.12	0.07	0.001, 0.14	0.19	0.12	0.05, 0.19
High-middle	0.11	0.05	−0.002, 0.10	0.19	0.08	0.03, 0.13
High	–		–	–		–
Age	−0.15	−0.02	−0.04, −0.01	−0.06	−0.01	−0.02, 0.004
Pre-pregnancy BMI	0.21	0.03	0.02, 0.04	0.28	0.04	0.03, 0.05

*Note:* BMI = body mass index.

* Variable measured at EAT-I.

## Data Availability

Data will be made available on request.

## Footnotes

1 The authors acknowledge that not all pregnant individuals identify as women. As such, we have elected to use gender-neutral terms like “birthing people” and “pregnant people” throughout this manuscript to reflect this truth and support inclusivity.

2 We use the term “structurally racialized groups” to acknowledge that race is neither biological nor objective but is rather a social construct.
